# A Non-inferiority, Randomized Clinical Trial Comparing Paclitaxel-Coated Balloon Versus New-Generation Drug-Eluting Stents on Angiographic Outcomes for Coronary De Novo Lesions

**DOI:** 10.1007/s10557-021-07172-4

**Published:** 2021-03-13

**Authors:** Xue Yu, Xinyue Wang, Fusui Ji, Wenduo Zhang, Chenguang Yang, Feng Xu, Fang Wang

**Affiliations:** Department of Cardiology, Beijing Hospital, National Center of Gerontology; Institute of Geriatric Medicine, Chinese Academy of Medical Sciences, 100730 Beijing, P. R. China

**Keywords:** Drug-coated balloon, Coronary artery disease, De novo lesion, Drug-eluting stent, Angioplasty

## Abstract

**Background:**

Drug-coated balloon (DCB) has been proved efficacy for coronary small vessel disease, but data regarding outcomes of DCB in common de novo lesions (including reference vessel diameter more than 3.0mm) compared with new-generation drug-eluting stent (DES) are lacking. We hypothesized that a DCB-only strategy for coronary de novo lesions would be non-inferior to DES treatment on angiographic outcomes.

**Methods:**

In this randomized controlled trial, we compared the effect of DCB with DES on late lumen loss (LLL) at 9-month angiographic follow-up and 12-month major adverse cardiac events (MACEs), including death, non-fatal myocardial infarction, target lesion revascularization (TLR), and target vessel revascularization (TVR).

**Results:**

From July 2017 to July 2018, 288 consecutive patients with reference vessel diameter (RVD) between 2.25 and 4.0mm were screened. After proper pre-dilation, 170 patients were enrolled and randomized to the DCB and the DES groups at 1:1 ratio. Seven patients withdrew the consent forms during hospital stay (1 in DCB group, 6 in DES group). Two patients in DCB group underwent bailout stenting due to severe dissection after DCB release. The primary endpoint of 9-month LLL was −0.19±0.49mm with the DCB versus 0.03±0.64mm with the DES. The one-sided 97.5% upper confidence limit of the difference was −0.04mm, achieving non-inferiority of the DCB compared with the DES (*P*=0.019). The 12-month cumulative MACE rate was similar in the DCB and DES groups (2.44% vs. 6.33%, *P*=0.226).

**Conclusions:**

In this prospective study, the DCB only strategy for de novo lesion was non-inferior to the new-generation DES in terms of 9-month late lumen loss.

**Supplementary Information:**

The online version contains supplementary material available at 10.1007/s10557-021-07172-4.

## Introduction

Drug-coated balloon (DCB) is recommended treatment for in-stent restenosis [1] and also a valid treatment for de novo small vessel disease (SVD) [2–4]. DCB allow rapid and uniform release of anti-proliferative drugs throughout the lesion, which can inhibit neointimal hyperplasia and stimulate vascular endothelial healing [5]. Therefore, it may be an alternative to drug-eluting stent (DES), especially for its potential advantages of shorter (1–3 months) period of double anti-platelet therapy (DAPT), relatively simple to perform, less contrast, and leaving no metal residues. It had been proved that paclitaxel DCB alone was non-inferior to the second-generation DES in the treatment of coronary SVD angiographically and clinically [6, 7]. Our previous data showed that treatment of large coronary de novo lesions (reference vessel diameter (RVD) >2.8 mm) with DCB only was as safe and effective as using for SVD [8]. However, limited and inconsistent data are available for DCB in de novo lesions [9–11]. We conducted this study to determine DCB is non-inferior to the new-generation DES for de novo lesions in terms of angiographic late lumen loss (LLL).

## Materials and Methods

### Study Design and Patient Enrolment

**Fig. 1:**
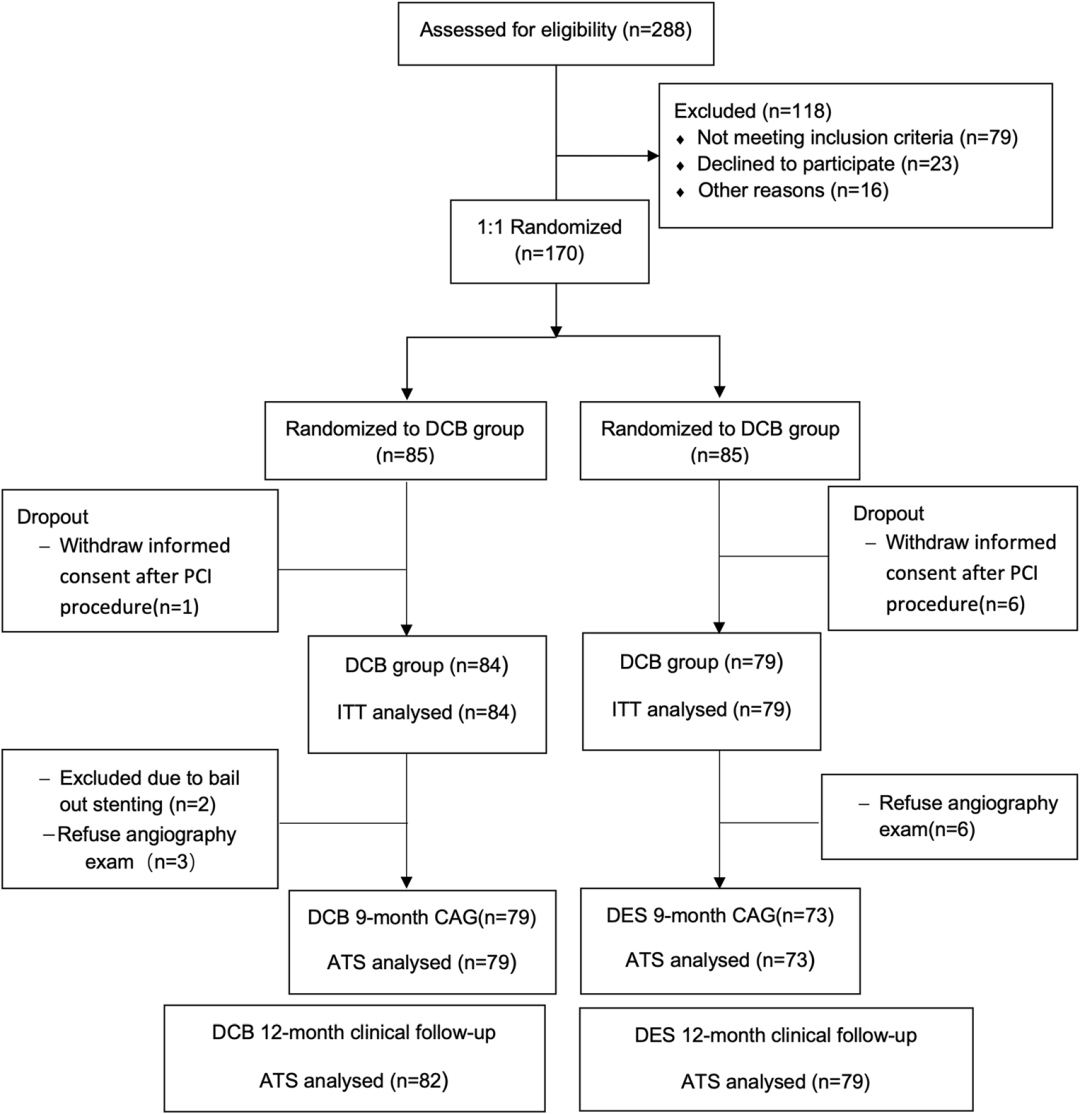
Study flow chart. DCB, drug-coated balloon; DES, drug-eluting stent

This prospective, randomized, open-label, single-center study was designed to evaluate the efficacy and safety of DCB in the treatment of coronary de novo lesions. Patients who underwent elective percutaneous coronary intervention (PCI) at Beijing Hospital were screened. The target lesions should not be intervened before, with a RVD between 2.25 and 4.0 mm and lesion length of ≤30 mm. The patients were consecutively enrolled and randomized into DCB and DES groups at a 1:1 ratio if the pre-dilation achieved ideal results (residual stenosis ≤30%, TIMI 3 flow, no dissection at the lesion or type A or B dissection [12], or type C dissection without blood flow restriction). The DCBs used were paclitaxel-coated (Sequent® Please; B/Braun Melsungen AG, Berlin, Germany), while the DES group received new-generation zotarolimus-eluting (Resolute Integrity; Medtronic, Santa Rosa, CA; 30/79), everolimus-eluting (Xience Xpedition; Abbott Vascular, Santa Clara, CA; 27/79; or SYNERGY; Boston Scientific Corporation, Marlborough, MA; 7/79), or rapamycin-eluting stents (Firehawk, MicroPort, Shanghai, China; 15/79). The main exclusion criteria included acute myocardial infarction (MI) within 1 week, left ventricular ejection fraction <40%, chronic total occlusion, left main disease, or multiple vessel disease with more than one lesion requiring treatment. Detailed inclusion and exclusion criteria are listed in the Supplement file. The study flowchart is shown in Fig. 1.

### Endpoints and Definitions

The primary endpoint was the lumen loss (LLL) of target lesions at the 9-month angiographic follow-up. LLL was defined as the minimal lumen diameter (MLD) immediately after the procedure minus the MLD at 9 months. The secondary endpoint was the major adverse cardiovascular events (MACE) after 12 months. MACE was defined as the composite of cardiac death, non-fatal myocardial infarction, target vessel revascularization (TLR), and target vessel revascularization (TVR). Cardiac death was defined as any death that was not clearly of extracardiac origin, and myocardial infarction, according to guidelines. Myocardial infarction was defined as evidence of myocardial necrosis consistent with the third International Definition of Myocardial Infarction [13]. TVR was defined as any repeat revascularization of the target vessel, and TLR was defined as any repeat revascularization within the stented or DCB-treated segment. Periprocedural MI was defined as an elevation of cardiac biomarkers (troponin or creatine kinase-myocardial band) >3 times the upper limit of normal. Stent thrombosis was classified according to the Academic Research Consortium definition [14]. Device success was defined as the ability of the investigational device to be delivered, dilated, and retrieved from the target lesion.

### Interventional Procedures

PCI was done under DAPT with aspirin (100 mg/day) and clopidogrel (75 mg/day) or ticagrelor (90 mg twice a day). Before PCI, 70–100 U/kg of unfractionated heparin was administered, with an additional 1000 U for every extra hour of procedural time. Glycoprotein IIb/IIIa antagonists were administered at the operator’s discretion. Non-target vessel lesions were to be treated before the target vessel lesion intervention with no complications. Otherwise, the patients were excluded. Patients in the DCB group with stable coronary disease received DAPT for 1–3 months [15] or for 6–12 months with acute coronary syndrome after PCI. Patients in the DES group received standard12-month DAPT.

Appropriate pre-dilation was performed before using a paclitaxel-coated balloon catheter with a balloon/vessel diameter ratio of 0.8–1.0, according to the recommendations of the German [15] and Chinese consensus groups [16]. Non-compliant (NC), cutting, scoring, or non-slip element (NSE) balloons were used for dilation to reduce severe intimal dissection. The DCB/vessel diameter ratio was also 0.8–1.0, and both ends of the balloon extended beyond both margins of the lesion by 2–3 mm under a pressure of 7–10 atm for 30–60 s. Each DCB catheter was only released once. Technical success was defined as residual stenosis ≤30% on quantitative coronary angiography (QCA) and grade 3 TIMI flow. If an apparent dissection (i.e., type C or above) occurred and the TIMI flow was below grade 3, the intervention was considered a failure and bailout stenting with DES was performed. Patients in the DES group were stented according to standard practices and the operator determined whether post-dilation was needed.

### Randomization and Masking

A total of 170 eligible patients enrolled were randomized to receive the DCB or DES in a 1:1 ratio using a random number. Number randomization was performed by a statistician using SPSS 22.0 software (IBM, Munich, Germany). This trial was open labeled; therefore, participants or investigators were not masked to the treatment.

### QCA

All angiograms were carefully recorded in all critical periods. At least 2 orthographic views (reference views) were required in pre-procedural angiograms at lesion site, accurate DCB location angiograms obtained before dilation, and 2 post-procedural angiograms with a similar projection angle as the pre-procedural angiograms. Follow-up angiograms were recorded with a similar projection angle as the post-procedural angiograms. All lesions were analyzed using the software built in Allura Xper FD20 flat-panel digital cardiac system (Philips Healthcare, Amsterdam, Netherlands). Two independent technicians who were not involved in the intervention measured and recorded the RVD, MLD, lesion length, percent diameter stenosis (DS%), and percent area stenosis. Measurements were performed in triplicate and the mean value was recorded.

### Statistical Methods

Data processing and statistical analyses will be performed using the IBM SPSS Statistics, version 22.0 (IBM Corp, Armonk, NY). Sample size calculation is based on the primary end point of the study, the LLL measurement during coronary angiography at 9-month follow-up. This trial is powered to show non-inferiority of a DCB only strategy versus DES for coronary de novo lesions. According to the previous studies, the mean LLL of DCB and new-generation DES was about 0.06mm [2, 17–19] and 0.14mm [20–22] respectively; the average standard deviation of 9-month LLL of paclitaxel DCB or the DES was approximately 0.50 mm [17–22]. For the sample size calculating, we wanted to achieve 90% power to detect non-inferiority using a one-sided, two-sample *t-*test with a one-sided *P* value <0.025. Non-inferiority for in-device late loss was declared if the upper limit of the one-sided 95% confidence interval (CI) difference in late loss (DCB minus DES) did not exceed a delta of 0.15 mm from the observed late loss in the DES group according to results reported previously [2, 4, 23]. At least 72 patients per group are needed to meet these criteria. Assuming the drop-out rate was 15%, at least 85 patients per group should be enrolled. A total of 170 patients will be randomized to provide sufficient power to achieve primary end point. The study is not powered for secondary endpoints. The baseline statistical analyses followed intention-to- treat (ITT) principles. The as-treated set (ATS) analyses were used in the items of QCA calculation and clinical follow-up (Fig. 1).

Data were processed using SPSS 22.0 (IBM, Munich, Germany). Continuous variables were tested for normality and were expressed as $\overline{x}\pm s$ in case of normal distribution or as median (interquartile range) if not normally distributed. Continuous variables were compared by the independent *t*-test or Mann-Whitney *U* test. Categorical data were expressed as rates or percentages. Inter-group comparisons were performed by the *χ*^2^ test. Fisher’s exact probability test was used when the theoretical frequency (T) was <5. Two-sided tests were used, with *P* <0.05 indicating a statistically significant difference.

## Results

### Patient Characteristics

From July 2017 to July 2018, 170 patients were enrolled and randomized to DCB or DES group at 1:1 ratio. Seven patients withdraw their consent forms after PCI procedure; therefore, 84 patients in DCB group and 79 patients in DES group were analyzed for baseline data. The demographic and baseline clinical data of the two groups were well matched. Unstable angina pectoris was the most common disease type in both groups (69.0% vs. 70.9%). There was no difference in the number of diseased vessels per patient, with >80% of both groups having multivessel (≥2) disease. Patients with three-vessel disease were slightly more common in the DES group (54.4% vs. 40.5%), but the difference was not significant (*P*=0.075) (Table 1)

**Table 1:** Patient baseline characteristics

	DCB group (*n*=84)	DES group (*n*=79)	Statistical value	*P* value
Demographic data				
Mean age (years)^a^	62.6±8.8	64.0±10.5	1.553	0.214
Male (%)	62 (73.8)	56 (70.9)	1.175	0.676
BMI^a^	26.0±3.0	25.4±3.0	0.025	0.874
Medical history				
Smoking (%)	46 (54.8)	42 (53.2)	0.042	0.838
Diabetes (%)	16 (19.0)	23 (29.1)	2.266	0.132
Hypertension (%)	50 (59.5)	54 (68.4)	1.375	0.241
Dyslipidemia (%)	52 (61.9)	39 (49.4)	2.595	0.107
Previous CABG (%)	1 (1.2)	3 (3.8)	1.156	0.355
Previous PCI (%)	11 (13.1)	14 (17.7)	0.671	0.413
History of atrial fibrillation (%)	2 (2.4)	5 (6.3)	1.544	0.266
History of cardiac insufficiency (%)	4 (4.8)	7 (8.9)	1.087	0.297
Type of coronary heart disease			0.943	0.815
Stable angina pectoris (%)	8 (9.5)	10 (12.7)	0.407	0.523
Unstable angina pectoris (%)	58 (69.0)	56 (70.9)	0.065	0.798
NSTEMI (%)	13 (15.5)	9 (11.4)	0.582	0.446
STEMI (%)	5 (6.0)	4 (5.1)	0.062	1.000
Distribution of coronary lesions			3.456	0.178
One-vessel disease (%)	14 (16.7)	12 (15.2)	0.066	0.797
Two-vessel disease (%)	36 (42.9)	24 (30.4)	2.725	0.099
Three-vessel disease (%)	34 (40.5)	43 (54.4)	3.181	0.075

*DCB*, drug-coated balloon; *DES*, drug-eluting stents; *BMI*, body mass index; *CABG*, coronary artery bypass graft; *PCI*, percutaneous coronary intervention; *NSTEMI*, non-ST-segment elevation myocardial infarction; *STEMI*, ST-segment elevation myocardial infarction

^a^Expressed by $\overline{x}\pm s$, while the rest of the data is expressed as number (%); The statistical result for the counted data refers to the chi-square test results, while that for the measurement data refers to the *t*-test results

### Lesion and Procedural Characteristics

The average target lesion RVD was approximately 3.0 mm [2.77 (2.50 to 3.25) mm vs. 3.01 (2.65 to 3.39) mm *P*=0.09], and large vessel disease (LVD; RVD ≥3.0 mm) accounted for 40.5% and 54.4% of cases, respectively (*P*=0.075). The proportion of calcified, tortuous, and type B2/C lesions was similar in both groups. The target lesion length in the DES group was similar with that in the DCB group.

The proportion of specialized balloons used to achieve effective pre-dilation was similar in both groups. The diameter of the DCB finally implanted [2.75 (2.50 to 3.00) mm] was significantly lower than that of the DES [3.00 (2.75 to 3.50) mm] (*P*<0.001), and the ratio of the implanted device diameter to the RVD was also significantly different [0.98 (0.86 to 1.04) vs. 1.01 (0.93 to 1.09), *P*=0.025] despite the similar pre-procedural RVD. A certain amount of residual stenosis was allowed after DCB treatment (33.20 ± 12.07% vs. 19.38 ± 7.80%, *P*<0.001) (Table 2).

**Table 2:** Lesions baseline data and procedural characteristics

	DCB group (*n*=84)	DES group (*n*=79)	Statistical value	*P* value
Target vessel			9.892	0.007^*^
LAD/D (%)	48 (57.1)	35 (44.3)	2.685	0.101
LCX/OM (%)	24 (28.6)	16 (20.3)	1.521	0.217
RCA/PDA, PL (%)	12 (14.3)	28 (35.4)	9.841	0.002
Feature of lesions				
RVD (QCA) (mm)	2.77 (2.50 to 3.25)	3.01 (2.65 to 3.39)	−1.697	0.09
RVD ≥3.0 mm (%)	34 (40.5)	43 (54.4)	3.181	0.075
Tortuous lesion (%)	26 (31.0)	31 (39.2)	1.230	0.267
Calcified lesion (%)	11 (13.1)	12 (15.2)	0.147	0.701
Type B2/C lesion (%)	39 (46.4)	33 (41.8)	0.358	0.550
Diameter stenosis (visual)	0.75 (0.75 to 0.90)	0.90 (0.75 to 0.90)	−1.620	0.105
Diameter stenosis (QCA)	0.61 (0.54 to 0.79)	0.67 (0.56 to 0.78)	−1.033	0.302
Pre-intervention MLD (mm)	1.06 (0.61 to 1.36)	1.00 (0.54 to 1.39)	−0.355	0.722
Lesion length (mm)	18.2 (16.0 to 20.1)	20.0 (15.0 to 25.0)	−1.277	0.202
Procedure data				
Maximum pre-dilation balloon diameter (mm)	2.50 (2.50 to 3.00)	2.50 (2.50 to 3.00)	−1.462	0.144
Combined special balloon (%)	39 (49.4)	40 (47.6)	0.050	0.823
Pre-dilation balloon diameter/RVD ratio	0.93 (0.83 to 1.01)	0.94 (0.83 to 1.01)	−0.208	0.836
Diameter of device finally implanted (mm)	2.75 (2.50 to 3.00)	3.00 (2.75 to 3.50)	−4.600	<0.001
Maximum device expansion pressure (atm)	8.0 (8.0 to 10.0)	10.0 (10.0 to 12.0)	−8.678	<0.001
Device inflation time (s)	40.0 (30.5 to 45.0)	10.0 (9.0 to 11.0)	−10.939	<0.001
Implanted device diameter/RVD ratio	0.98 (0.86 to 1.04)	1.01 (0.93 to 1.09)	−2.244	0.025
Implanted device length (mm)	17.50 (15.0 to 20.0)	23.0 (18.0 to 28.0)	−4.358	<0.001
Dissections after device released (%)	23 (27.4)	4 (5.1)	14.672	<0.0001
			16.704	0.001 (constitution)
Type A (%)	6 (7.1)	3 (3.8)	0.873	0.497
Type B (%)	9 (10.7)	1 (1.3)	6.311	0.012
Type C (%)	8 (9.5)	0	7.912	0.007
Type D and above (%)	0	0	-	-
Residual DS%	33.2±12.1	19.4±7.8	9.147 (8.621)	<0.001
Bailout stenting (%)	2 (2.4)	0 (0)	1.904	0.497
Device success rate (%)	97.6	98.7	0.280	1.000

*DCB*, drug-coated balloon; *DS%*, percentage diameter stenosis; *RVD*, reference vessel diameter; *QCA*, quantitative coronary angiography; *DES*, drug-eluting stents; *LAD/D*, left anterior descending/diagonal branch; *LCX/OM*, left circumflex/obtuse marginal branch; *RCA/PDA/PL*, right coronary artery/posterior descending artery/posterior lateral. The measurement data is expressed as $\overline{x}\pm s$ or median (standard deviation), and the rest as *n* (%). The statistical results for the counted data refer to the chi-square test results, while that for the measurement data refers to the *t*-test results

Dissection after device release was observed in 27.3% of DCB cases, significantly higher than after DES. Most dissections were types B or C. Only 2 patients with type C dissection underwent bailout stenting when the blood flow decreased to TIMI grade 2 during the in-lab observation period (5–10 min after DCB deployment). The procedural success rate in the DCB group was 97.6% (82/84), similar to that in the DES group 98.7% (78/79).

### Angiographic Results Immediately Post-intervention and During Follow-up

There was no difference in pre-intervention MLD between the two groups, but the post-intervention MLD (1.82±0.43mm vs. 2.54±0.50mm) and the immediate lumen gain (0.84±0.56mm vs. 1.53±0.64mm) were significantly lower in the DCB group than those in the DES group (both *P* <0.001).

**Fig. 2:**
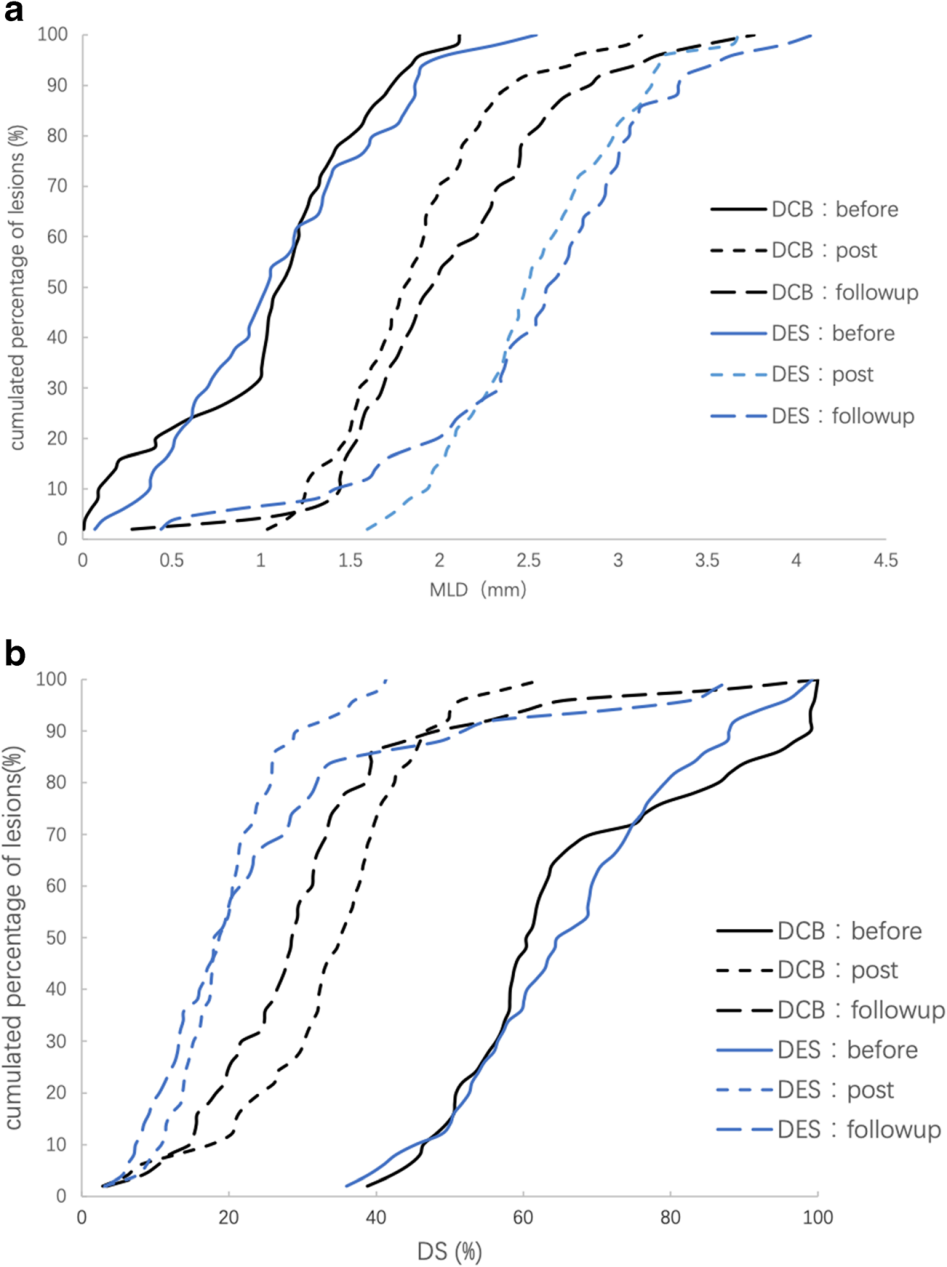
**a** Frequency distribution of MLD at the 9-month angiographic follow-up. **b** Frequency distribution of DS% at the 9-month angiographic follow-up. DCB, drug-coated balloon; DES, drug-eluting stent; MLD, minimal lumen diameter; DS% percentage of diameter stenosis

A total of 152 patients (79 in the DCB group and 73 in the DES group) completed angiographic follow-up at an average of 9 months. Two patients were dropped-out due to DCB failure and 9 patients refused angiographic follow-up due to lack of symptoms, financial difficulties, or fear of invasive procedures. The angiographic success rate was 94.4% (152/161). The 9-month follow-up MLD in the DCB group was significantly increased compared with post-intervention level (2.02±0.62mm vs. 1.83±0.44mm, *P* <0.001), while this trend was not observed in the DES group (2.49±0.76mm vs. 2.52±0.47mm, *P*=0.705). The primary endpoint of 9-month LLL was −0.19±0.49mm with the DCB versus 0.03±0.64mm with the DES. The 95% CI of the difference was −0.40mm to −0.04mm, achieving non-inferiority of the DCB compared with the DES (*P*=0.019). Comparing with those in the DES group, the follow-up MLD was significantly lower and the DS% was more severe in the DCB group, which was considered due to the lack of supporting structure after DCB delivery. However, the restenosis rate (defined as DS% ≥50%) was similar between the DCB and the DES groups (8.9% vs. 9.6%, *P*=0.877) (Table 3 and Fig. 2).

**Table 3:** Comparisons of pre-intervention and follow-up luminal diameter indices between the two groups

	DCB group (*n*=82)	DES group (*n*=79)	Statistical value	*P* value/difference (95% CI)
Lesions of enrolled patients				
Pre-intervention MLD (mm)	1.01±0.55	1.01±0.59	−0.023	0.982
Post-intervention MLD (mm)	1.82±0.43^#^	2.54±0.50^#^	−9.761	<0.001
Immediate lumen gain (mm)	0.85±0.56	1.53±0.64	−7.206	<0.001
9-month follow-up	* DCB group(n*=79)	*DES group(n*=73)		
Pre-intervention MLD (mm)	1.03±0.55	1.07±0.57	−0.433	0.666
Post-intervention MLD (mm)	1.83±0.44^#^	2.52±0.47^#^	−9.328	<0.001
Immediate lumen gain (mm)	0.81±0.58	1.45±0.52	−7.137	<0.001
Follow-up MLD (mm)	2.02±0.62^*^	2.49±0.76	−4.216	<0.001
LLL (mm)	−0.19±0.49	0.03±0.64	−2.363	0.019/−0.22 (95% CI: −0.40 to −0.04)
Pre-intervention DS, %	60.4 (53.8 to 78.6)	64.6 (54.2 to 76.5)	−0.549	0.583
Post-intervention DS, %	35.1 (26.5 to 40.4)	18.7 (14.3 to 23.7)	−7.295	<0.001
Follow-up DS, %	28.5 (20.0 to 34.3)	18.0 (12.3 to 29.3)	−3.719	<0.001
Restenosis lesion (%)	7 (8.9)	7 (9.6)	0.024	0.877

*DCB*, drug-coated balloon; *DES*, drug-eluting stents; *MLD*, minimal lumen diameter; *LLL*, late luminal loss; *DS*, diameter stenosis. ^#^Compared with pre-intervention MLD, *P* <0.001; ^*^Compared with post-intervention MLD, *P* <0.001

### Thirty-Day and 12-Month Clinical Follow-up

There were no deaths or MIs during hospital stay in the DCB group. One patient in the DES group developed thromboembolism at the distal end of the second obtuse marginal branch after stenting of the proximal left circumflex artery and was diagnosed perioperative MI according to the increase in myocardial enzymes. No further intervention was performed due to the very small vessel diameter.

All the 161 patients completed the 12-month clinical follow-up. One MI had occurred in each group. There were no significant differences in target vessel MI, TLR, TVR, cardiac death, and all-cause death between the two groups at 12 months. The MACE rate was 2.44% (2/82) and 6.33% (5/79) respectively, showing no significant difference (*P*=0.226) (Table 4).

**Table 4:** Clinical follow-up at 30 days and 12 months in the DCB and DES groups

Endpoint event	DCB group (*n*=82)	DES group (*n*=79)	Statistical result	*P* value
30 days				
Composite endpoint	0	1 (1.27)	1.070	0.485
Death	0	0	—	—
Non-fatal myocardial infarction	0	1 (1.27)	1.070	0.485
TVR	0	0	—	—
TLR	0	0	—	—
12 months				
Composite endpoint	2 (2.44)	5 (6.33)	1.464	0.271
Death	0	0	—	—
Non-fatal myocardial infarction	1 (1.22)	1 (1.27)	0.001	1.000
TVR	1 (1.22)	1 (1.27)	0.001	1.000
TLR	1 (1.22)	3 (3.80)	1.104	0.361

Values are expressed as *n* (%)

*DCB*, drug-coated balloon; *DES*, drug-eluting stents; *TVR*, target vessel revascularization; *TLR*, target lesion revascularization

## Discussion

The 9-month angiographic results showed that a DCB-only strategy for coronary de novo lesions was non-inferior to DES in terms of LLL. Furthermore, the 12-month composite MACE rates between the two groups were not significantly different.

Coronary de novo lesions are the most common lesions encountered in daily interventional practice, including both SVD and LVD. In this study, the median RVD was 2.95 mm, and patients with RVD ≥3.0 mm comprised 47.2% of our cohort. Previous studies on DCB only treatment of coronary de novo lesions have been mostly observational. Valentines II trial [17] was a prospective, multi-center registry study of DIOR® paclitaxel DCB for the treatment of early coronary de novo lesions. The study enrolled 103 patients (109 lesions) with RVD of 2.40 ± 0.51 mm, but the proportion of LVD was unknown. The 6-month angiographic follow-up showed that LLL of the target lesions was 0.38 ± 0.39 mm. Our group retrospectively analyzed 595 coronary de novo lesions treated with Sequent Please® paclitaxel DCB only. The average RVD was 2.48 ± 0.33 mm and LVD with RVD ≥2.8 mm accounted for 37.3% of all lesions. The 10-month angiographic follow-up showed LLL of (−0.17) ± 0.53 mm, with a TLR rate of 0.4% [8]. Two recent prospective observational studies reported 8-month LLL after DCB intervention for LVD of (−0.02) ± 0.49mm [10] and 0.01 ± 0.52 mm [24] respectively, suggesting that DCB is efficacious in de novo lesions. Nishiyama et al. [11] randomized 60 patients to DCB or DES after acceptable pre-dilation, but observed no significant difference in MLD and LLL at 8 months. In this study, a trend towards positive luminal remodeling (late lumen catch-up phenomenon) was noticed in the DCB group, which achieved non-inferiority to DES.

The recently published RESTORE SVD China study used DS% as the primary end point [7]. The authors argued that follow-up DS% was equally effective as luminal loss in predicting TLR, whereas the impact of LLL on the likelihood of TLR varies with vessel size. In the present study, the DS% of the DCB and DES groups at 9-month follow-up [28.5 (20.0 to 34.3) vs. 18.0 (12.3 to 29.3), *P*<0.001] were close to those in RESTORE SVD China (29.3 ± 20.2% vs. 22.8 ± 15.3%, *P*=0.01). When discussing the surrogate endpoints for DES clinical trials, Pocock et al. pointed out that the TLR rate would be very low if DS% in the stented segment was <30% at follow-up, and a lower DS% would not further reduce adverse clinical events [25]. Whether this notion is also applicable to DCB trials remains to be verified. TLR mainly depends on the severity of restenosis. When we defined restenosis as DS% >50% in this study, we found no significant difference in its incidence between the treatment groups. Correspondingly, there were no significant differences in TLR either.

There was no difference in the 12-month clinical endpoint between the two groups in this study, which was consistent with the non-inferior DCB angiographic results. Similar results have also been reported in recent observational studies. In the DEBATE study [9], 120 patients with coronary heart disease (135 de novo lesions) and RVD of 3.09 ± 0.31 mm were treated with Sequent Please® paclitaxel DCB. Two patients (1.6%) underwent bailout stenting and 4 (3.4%) received TLR at 12 months, but no cardiac deaths, MIs, or TVRs occurred. Similarly, two other prospective studies reported TLR and MACE rates of 3.9% [24] and 4.3% [10] respectively, with no MIs or deaths. The BASKET-SMALL2 randomized multi-center clinical trial (758 patients) showed that the Sequent Please® DCB was noninferior to first-/second-generation DES (Taxus, Xience Prime) in the treatment of de novo SVD (RVD <3 mm) [6]. Although our sample size insufficient to determine a difference in clinical endpoints, the angiographic results implied that patients might benefit from DCB treatment.

The key to successful DCB treatment for coronary de novo lesions is achieving desirable pre-dilation. Most consensus statements [15, 16, 26] recommend a pre-dilation balloon diameter/RVD ratio of 0.8–1.0:1. The newly published Third Report of the International DCB Consensus recommended balloon-to-vessel ratio should be 1:1 [27]. Given heavy plaque load or significant calcification and fibrosis, cutting or scoring balloons could be used in combination with NC balloons to avoid severe dissection. In this study, the maximum pre-dilation balloon diameter/RVD ratio in both groups was about 0.93, suggesting effective pre-dilation. The final DCB/RVD and DES/RVD ratio was 0.98 (0.86 to 1.04) and 1.01 (0.93 to 1.09) (*P*=0.025) respectively, suggesting that the angiographic success criteria for the two procedures are different. The main function of DCB is to deliver the drug to the lesion and transfer it into the vessel wall [28], rather than expanding the lumen. To avoid technical failure, an excessively large DCB or release at a pressure significantly above the nominal pressure should be avoided. Residual stenosis ≤30% is acceptable. DES, however, must be fully expanded and well adherent to the vessel wall to reduce the incidence of in-stent thrombosis or restenosis.

Hermans et al. showed that after successful POBA (residual stenosis <50%), the restenosis rate was 29% in patients with dissection and 30% in those without dissection, and dissection did not increase the incidence of MACE [29]. Type C dissection after DCB deployment was observed in 8 (9.5%) lesions in our study, 6 of which were not treated with stents as the dissection did not progress after 10–15 min of observation, while 2 required stenting due to impaired distal blood flow. No in-hospital complications, such as acute vascular occlusion or thrombosis, occurred in the DCB group. All lesions with dissection (21/84) had healed at the 9-month angiographic follow-up, similar to previous reports [8, 9]. This form of dissection was termed “therapeutic dissection” [29], which does not significantly compress the coronary lumen, affect blood flow, or cause adverse events, allowing patients to be discharged as scheduled. Studies have shown that restenosis of this dissection type is uncommon [30–32]; however, more angiographic follow-up data is required to determine if dissection after DCB is beneficial for positive vascular remodeling.

## Limitations

There were several limitations in this study. First, the study was powered for the primary endpoint but was not insufficient to verify the differences in clinical endpoint events. Second, single-center study would introduce bias. Third, the DAPT duration in the DCB group showed great variability, with patients receiving treatment for 3 months if they had no previous PCIs or PCI more than 12 months before enrollment, while others received DAPT for 3–12 months if they had undergone DES implantation within 12 months of enrollment. This heterogeneity may affect the clinical endpoint. Fourth, we did not restrict the type or number of balloons used for pre-dilation and post-dilation since physicians have their own preferences. We also did not specify the stent brand, considering that the efficacy of the new-generation DES used at our center seemed equivalent; however, differences may exist.

## Conclusions

This prospective single-center randomized study showed that paclitaxel DCB only strategy for coronary de novo lesions is noninferior to the new-generation DES in term of LLL at 9 months. Moreover, there was no significant difference in MACEs rate at 12 months.

## Supplementary Information

ESM 1 (DOCX 27 kb)

## Data Availability

All participants were enrolled at the Department of Cardiology in Beijing Hospital. The data underlying this article will be shared on reasonable request to the corresponding author.

## Code Availability

All statistical analyses were generated with Statistical Package for Social Sciences (IBM SPSS version 22.0, Armonk, New York, USA). All the target coronary lesions were analyzed using the software built in Allura Xper FD20 flat-panel digital cardiac system (Philips Healthcare, Amsterdam, Netherlands).
