# Supplementary material for: A Non-inferiority, Randomized Clinical Trial Comparing Paclitaxel-Coated Balloon Versus New-Generation Drug-Eluting Stents on Angiographic Outcomes for Coronary De Novo Lesions
Source: Cardiovasc Drugs Ther. 2021 Mar 13;36(4):655–64. doi: 10.1007/s10557-021-07172-4 (PMC9270292; doi:10.1007/s10557-021-07172-4)
Supplement: Supplementary file 1 — (DOCX 27 kb) [file 10557_2021_7172_MOESM1_ESM.docx]

**Supplement Table:** Eligibility Criteria

| Inclusion criteria:  *Patient-related:* |
| --- |
| - age between 18–80 years; - Stable angina or unstable angina; - previous myocardial infarction or proven asymptomatic ischemia; - agreeing to undergo coronary intervention and angiographic follow-up at 9 months - agreeing to undergo clinical follow-up at 30 days, 9 months, and 12 months.   *Lesion-related:*   - De novo coronary lesions with reference diameter is ≥ 2.25 mm and ≤ 4.0mm, and ≤ 30 mm long; - pre-operation diameter stenosis ≥ 70% or evidence of ischemia if < 70%; - no dissection or type A/B dissection, residual stenosis ≤ 30% and thrombolysis in myocardial infarction (TIMI) grade 3 flow after predilation; - distance between other lesions requiring interventional treatment and target lesions of >10 mm and had been treated before the target lesion intervention; - only one drug-coated balloon or drug-eluting stent to be used for each target lesion.   Exclusion criteria:  *Patient-related:*   - Acute myocardial infarction within one week, clinically stable and had not undergo primary PCI or urgent PCI; - severe congestive heart failure (LVEF < 40% or NYHA III/IV); - severe valvular heart disease; - pregnant or lactating women; - leukopenia or thrombocytopenia; history of peptic ulcer or gastrointestinal hemorrhage in the past 6 months; - history of severe liver and kidney failure; stroke within 6 months before procedure; - life expectancy of less than 1 year or difficulty in clinical follow-up; - susceptible to bleeding or contraindicated for anticoagulants or antiplatelets; - inability to tolerate aspirin and/or clopidogrel or known intolerance or allergic to heparin, contrast agent, paclitaxel, iopromide, rapamycin, polylactic acid-glycolic acid polymer, cobalt chromium alloy, or platinum chromium alloy.   *Lesion-related:*   - Extensive thrombus in the target vessel; - intervention in the bridging vessel; - chronic total occlusive lesions (pre-procedural TIMI flow grade 0); - left main disease; - multiple vessel disease (two or three vessels with lesions more than 50% stenosis) with more than one lesion was >75% stenosis or with definite ischemia evidence requiring treatment. |
